# Rho protein GTPases and their interactions with NFκB: crossroads of inflammation and matrix biology

**DOI:** 10.1042/BSR20140021

**Published:** 2014-06-25

**Authors:** Louis Tong, Vinay Tergaonkar

**Affiliations:** *Singapore National Eye Center, 11 Third Hospital Avenue, Singapore 168751; †Singapore Eye Research Institute, Singapore; ‡Duke-NUS Graduate Medical School, Singapore; §Yong Loo Lin School of Medicine, National University of Singapore, Singapore; ∥Institute of Molecular and Cell Biology, A*Star Institute in Singapore, Singapore

**Keywords:** cell biology, cell signalling, inflammation, NFκB, RhoGTPase, BCR, B-cell receptor, COX2, cyclo-oxygenase 2, EMT, epithelial–mesenchymal transition, GAP, GTPase-activating protein, GDI, guanosine-nucleotide-dissociation inhibitor, GEF, guanine-nucleotide-exchange factors, HIF, hypoxia-inducible factor, IFN, interferon, IKK, IκB, inhibitory κB, IL-1RAcP, interleukin 1 receptor accessory protein, JNK, c-Jun N-terminal kinase, LPS, lipopolysarrcharide, MAPK, mitogen-activated protein kinase, MEKK1, MEK (MAPK/ERK kinase) kinase 1, MMP, matrix metalloproteinase, NEMO, NFκB essential modulator, NFκB, nuclear factor κB, NIK, NFκB-inducing kinase, PAK, p21-activated kinase, ROCK1, Rho-associated protein kinase 1, ROS, reactive oxygen species, Tak1, TGF (transforming growth factor)-β-activated kinase 1, TSG, tumour-susceptibility gene, TANK, TRAF-associated nuclear factor κB activator, TLR, Toll-like receptor, TNFα, tumour necrosis factor α, TRAF, TNF-receptor-associated factor

## Abstract

The RhoGTPases, with RhoA, Cdc42 and Rac being major members, are a group of key ubiquitous proteins present in all eukaryotic organisms that subserve such important functions as cell migration, adhesion and differentiation. The NFκB (nuclear factor κB) is a family of constitutive and inducible transcription factors that through their diverse target genes, play a major role in processes such as cytokine expression, stress regulation, cell division and transformation. Research over the past decade has uncovered new molecular links between the RhoGTPases and the NFκB pathway, with the RhoGTPases playing a positive or negative regulatory role on NFκB activation depending on the context. The RhoA–NFκB interaction has been shown to be important in cytokine-activated NFκB processes, such as those induced by TNFα (tumour necrosis factor α). On the other hand, Rac is important for activating the NFκB response downstream of integrin activation, such as after phagocytosis. Specific residues of Rac1 are important for triggering NFκB activation, and mutations do obliterate this response. Other upstream triggers of the RhoGTPase–NFκB interactions include the suppressive p120 catenin, with implications for skin inflammation. The networks described here are not only important areas for further research, but are also significant for discovery of targets for translational medicine.

## RHO AND NFκB (NUCLEAR FACTOR κB): KEY CELLULAR REGULATORS

The Rho family of GTPases consist of 20 known ubiquitous proteins that regulate cell spreading, adhesion and movement [1]. RhoA, Rac and Cdc42 are the key members. Rho proteins cycle between active GTP-bound and inactive GDP-bound forms. The availability of GAPs (GTPase-activating proteins), GEFs (guanine-nucleotide-exchange factors) and GDIs (guanosine-nucleotide-dissociation inhibitors) regulate the activity of these proteins. Higher organisms have a complex machinery that regulates Rho proteins and their downstream effectors [2–4].

Critical tools used for probing Rho protein function along with their advantages and disadvantages, have been listed in Table 1. These include, among others, dominant negative mutants [5], and chemicals and toxins to covalently modify Rho proteins. Deamination of the proteins result in activation while ribosylation or glucosylation can inactivate them [6,7]. Since naturally occurring mutants of RhoGTPases have not been described, there is some doubts about the applicability of research using mutants. On the other hand, approaches based on overexpression of GEFs may be more valid, as GEFs such as Dbl and Ost have been isolated from different cancers, and Vav has been derived from a human haematopoietic cell line [8]. The disadvantage of this approach is that a particular GEF may interact with more than one RhoGTPase, leading to pleiotropic effects.

**Table 1:** Tools in Rho protein research

Tool	Basis and advantages	Disadvantages
Active RhoGTPase mutants, e.g. glutamine-to-leucine substitution at position 16 of rac (Rac1Q61L or Rac1-L61)	GTPase activity inactivated	Like all genetic active mutants, may not be physiologically relevant
	Constitutively bound to GTP	These activating mutations not found in human tumours
Dominant negative RhoGTPase mutants, e.g. serine-to-asparagine substitution at position 17 of rac (Rac1-N17)	Favour the GDP bound form of RhoGTPase, with	May not distinguish closely related rho members such as Cdc42 and Tc10
	Reduced affinity for GTP	
	Reduced availability of GEFs	
	Targeted to distinct subcellular compartments as the wild-type rho, rac and cdc42, ensuring some specificity	
Bacterial toxins, e.g. *Clostridium difficile* (from which the cytotoxins A and B are derived) or *Clostridium botulinum* (from which the C3 transferase is derived)	Cytotoxins A and B are cation-dependent UDP-glucose glucosyltransferases	Useful to screen for the involvement of Rho proteins only
	Inactivate RhoA, Rac and Cdc42 through monoglucosylation using UDP-glucose as a co-substrate.	May not have been tested on all Rho proteins
	Some specificity for *Clostridum difficile* cytotoxins A and B:	
	Small GTPases Ras, Rab, Arf or Ran and the large heterotrimeric G-proteins and are not modified by these toxins	
	Some specificity for C3 for RhoA, B and C	
Lovastatin	Deplete geranylgeranyl and farnesyl precursors	Probably not specific as rho inhibitor
	Inhibit isoprenylation	Not easy to determine dosage of use
	Localization of Rho to membranes requires C terminal isoprenylation [116,117]	
	Drug destroys the normal intracellular distribution of Rho and therefore its function [118,119]	

The NFκB pathway is a conserved signalling cascade involved in diverse physiological processes [9–14]. Hyperactivation of NFκB is linked to numerous human diseases and it is appreciated that the inactivation of NFκB, similar to its activation, also needs to be highly timed. Given that the temporal activation of NFκB is so critical, finding the various mechanisms that lead to constitutive NFκB activity in human ailments is very important [15]. Many stimuli, which include cell-surface ligands, inter-cytoplasmic and nuclear targets, lead to the activation of NFκB [16–18]. These stimuli share some common mechanisms of action in the initial and distal parts of the pathway.

Distally, the mechanism converges on the IKK [IκB (inhibitory κB) kinase] complex, consisting of IKK1, IKK2 and NEMO (NFκB essential modulator), which mediates the phosphorylation and degradation of IκB proteins. In addition, the complex also contains chaperones and adaptors such as ELKS and Rap1 [15]. Activation of the IKK complex in response to all stimuli is triggered by the phosphorylation of two key serine residues in their respective activation loops by the upstream kinase Tak1 [TGF (transforming growth factor)-β-activated kinase 1] [15]

In normal resting cells, cytosolic IκB binds and inhibits NFκB from translocating to the nucleus for target gene transcription. During activation of the canonical NFκB pathway, the NFκB transcription factor must be released from the IκB proteins. IκB is phosphorylated by IKK and then ubiquitinated by K-48 linked ubiquitin chains. These poly-ubiquitin tags are recognized by the regulatory structures in the proteasome cap, resulting in the degradation of IκB proteins with varied kinetics depending on the characteristics of the activating stimuli [19].

A highly integrated but distinct pathway from that described above is the non-canonical NFκB pathway [20]. The central activating kinase for this pathway is called the NIK (NFκB-inducing kinase), and the degradation of this kinase is the main regulatory step in the pathway [21]. A set of tumour necrosis factor superfamily members are known to activate this system. The non-canonical pathway is independent of NEMO [20], but involves non-canonical IKKs such as the TANK [TRAF (TNF-receptor-associated factor)-associated nuclear factor κB activator]-binding kinase 1 [22]. The non-canonical NFκB component p100 can undergo processing when activated [18]. Indeed only a few non-IκBα-dependent functions of IKK complex have also been reported [23,24].

## CONNECTING RHO AND NFκB

RhoGTPases and the NFκB pathway are critically involved in human diseases and may be potential therapeutic targets [25]. Distinct Rho proteins have been involved in positive or negative regulation of NFκB in different contexts (Table 2). NFκB activation can occur via a range of pleiotropic soluble and extracellular ligands, or intracellular stimuli related to DNA damage and ROS (reactive oxygen species). Both types of NFκB activators can be mediated by RhoGTPases, and even closely related RhoGTPases can be located in different subcellular locations [26].

**Table 2:** Mechanisms of NFκB activation by RhoGTPases

RhoGTPase or GEF	Type of regulation	NFκB component regulated by RhoGTPase signalling	Cell type evaluated
RhoA, Rac1, Rac2, Cdc42 and Rac1b	Positive	IκBα [41]	Simian COS-7, NIH 3T3 fibroblasts, human T-cell lymphoma Jurkat, rabbit synovial fibroblasts, human cervix carcinoma HeLa, IκBα participation not specifically investigated: human vascular endothelial HUVEC, human astrocytoma cell line U-373-MG, non-transformed human colonic epithelial cell NCM460
RhoA, Rac	Positive	Phosphorylation of p65, Association of p65 with NIK, Nuclear p52 processing [100]	HepG2 hepatocytes
RhoA	Negative	IκBα (nitric oxide pathway) [84]	C6 glioma
RhoB–F	Positive	Phosphorylation of p65 (endosomal pathway) [89]	HeLa, breast cancer T47D, COS-7
RhoB (fibroblasts)	Negative	IκBα [87]	NIH 3T3
RhoH	Negative	IκBα [86]	T293, Jurkat
Rac1	Negative	?IκBα (Nod2 pathway) [88]	Myelomonocytic, intestinal epithelial cells
Rac1	Positive	Expression of p50/p105 [99]	Colorectal DLD-2
Rac1	Positive	IKKα and β, phosphorylation of p65 [92]	Macrophages
Rac3	Positive	Nuclear coregulator [98]	HeLa
Rac	Positive	Expression of p65 [56]	Breast tumour cells
Vav1, Dbl, Ost	Positive	IκBα [8]	Haematopoietic cells (erythroid, lymphoid and myeloid lineages)
Vav1	?	Nuclear coregulator [97]	Jurkat, granulocytes (HL60), megakaryoblasts (UT7), bone marrow-derived mast cells (BMMC), Rat basophilic leukaemia (RBL-2H3)

**Figure 1:**
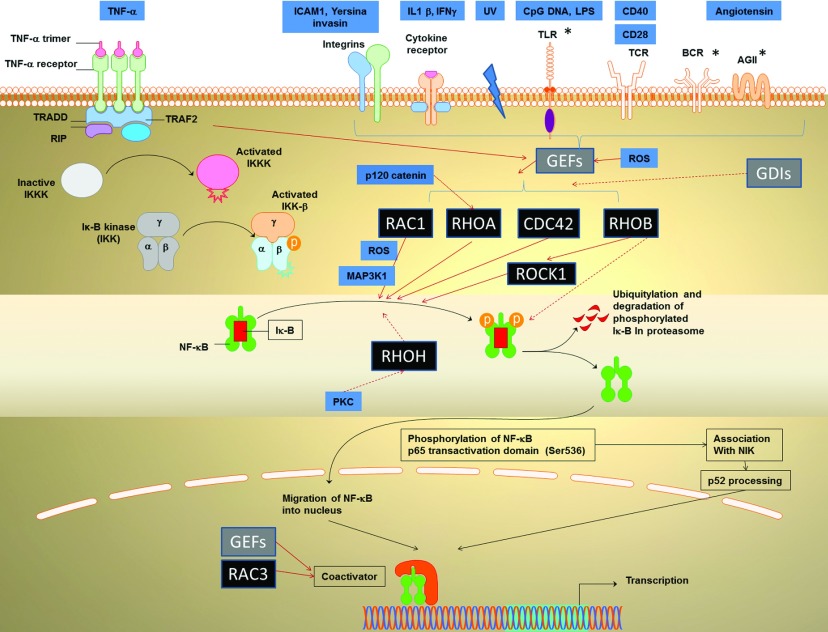
The classical paradigm of TNFα-induced RhoGTPase-activated NFκB signalling, mediated by degradation of the IκBα of the canonical NFκB pathway is shown on the left (black arrows) The possible link to non-canonical pathway is shown on the bottom right. RhoGTPases acting on this pathway are shown in black boxes. Grey boxes indicated regulatory elements for the RhoGTPases, while other regulatory molecules, including extracellular and intracellular molecules are shown in blue. The central regulatory point is the IκBα, but other regulatory points are also shown (see text for details). GEF, guanosine nucleotide exchange factors; GDI, guanosine-nucleotide-dissociation inhibitors. Positive regulation is shown by complete orange arrows and inhibition by broken orange arrows. PKC, protein kinase C; MAPK, mitogen-activated protein kinase; ROS, reactive oxygen species; IL, interleukin; IFN, interferon; ICAM, intercellular cell adhesion molecule; TRADD, tumour necrosis factor receptor type 1-associated DEATH domain protein; RIP, Receptor-interacting serine/threonine-protein kinase; TRAF, TNF receptor-associated factor; ROCK, Rho-associated protein kinase; BCR, B-cell receptor. *, these stimuli have been linked to p65 phosphorylation or non-canonical NFκB at least in certain scenarios.

Here we focus on various points along the NFκB pathway that can be regulated by the Rho family proteins (Figure 1), supporting this with a discussion of relevant work and findings with wider biological significance. Although there have been studies where NFκB activation in relation to RhoGTPases was discovered using reporter assays, the component of the NFκB involved was not specifically reported. For example, neurotensin [27] or substance P [28] was able to regulate IL-8 through Rho NFκB interaction, but the involvement of IκBα was not specifically investigated.

RhoGTPases are known to exert an effect on the cytoskeleton. NFκB may be a player in these processes or may be entirely separate from the cytoskeleton. The various roles that Rho proteins are involved in these pathways may be related to how different domains of RhoGTPases interact with downstream effectors [29].

For example, Rac1, which is a key RhoGPTase, has different domains interacting with respective effectors. The Rac1V12 H26 and Rac1V12 N130 mutants disrupt signalling domains for the effector PAK [p21 (Cdc42/Rac)-activated kinase], whereas Rac1V12 L37 disrupts cytoskeletal events without affecting the JNK (c-Jun N-terminal kinase) or PAK activation [30,31]. The expression of Rac1V12 L37, but not the H26 and N130, can disrupt NFκB activity compared with the un-mutated Rac1V12 [29]. These findings show that the Rac1 NFκB pathway may control actin organization. An unknown intermediate molecule related to cytoskeleton must interact with the Rac1 to exert its NFκB-activating effects.

## RHO GEFS AND RHO GDIS

It is now known that various upstream stimuli increase the activity of RhoGTPases (Figure 1). In almost all of these pathways, the RhoGTPase activity is enhanced due to increases in GEFs such as Vav 1, Vav 3, Dbl, Ost and GEFT (guanine-nucleotide exchange factor), rather than increased the expression of the RhoGTPases themselves. The GEF molecules have two regions, the DH (Dbl homology) and the PH (Pleckstrin homology) domains [32], which mediate GDP/GTP exchange. RhoGEFs are regulated by phosphorylation and they are known to interact with and be activated by the membrane receptors such as receptor tyrosine kinases, G-protein-coupled receptors and plexin-B [33,34]. Overexpression of Dbl, Vav or Ost can increase NFκB activity to similar extents as the overexpression of constitutively active RhoA, Rac1 and Cdc42. Different GEFs function to activate distinct Rho proteins.

Rho-GDIs, in contrast to GEFs, reduce NFκB activation. In C2C12 cells, expression of Rho-GDI resulted in reduction of mechanical strain-induced NFκB activation, suggesting that mechanical strain-induced NFκB requires RhoGTPases [35].

## PHOSPHORYLATION AND DEGRADATION OF IκBα (POSITIVE REGULATION OF NFκB)

Mechanisms of activating NFκB may or may not involve the IκB proteins [36,37]. Degradation of IκBα is the main regulatory point of the canonical NFκB pathway and most of the RhoGTPases-regulating NFκB are known to act on this molecule. It is not known if this may reflect the relative familiarity of researchers with this molecule and pathway, or that this is really the key point of input for RhoGTPases in NFκB.

ROS are the key intracellular stimulus that mediates the action of RhoGTPase on IκBα degradation and this operates at multiple points (Figure 1). Rac1 modulation of NFκB is mediated by ROS, and leads to the expression of inflammatory mediators: IL1 and collagenase-1, member of the MMPs (matrix metalloproteinases). [38] ROS are known to trigger inflammasome activation, resulting in caspase-1-mediated activation and release of IL1β [39]. Autocrine feedback through IL1 receptors then leads to further increased NFκB activation, and additional synthesis of IL1β (see the section on ‘stress-related pathways’). ROS is also involved in the NFκB activation via Vav1, a GEF for Rac [40]. The following subsections describe the regulation of RhoGTPases on IκBα degradation by extracellular stimuli:

### TNFα pathway

TNFα is a classic ligand that activates the NFκB pathway in a canonical fashion [20]. Elevated TNFα levels are a hallmark of many inflammatory diseases such as rheumatoid arthritis. It has been reported that the Rho proteins RhoA, Rac1 and Cdc42 play a role downstream of TNFα, resulting in NFκB activation [41].

When cells have been transfected with plasmids containing mutant IκBα that cannot be phosphorylated or degraded, mutant IκBα will compete with the endogenous IκBα to bind NFκB in the cytoplasm. Upstream activators such as RhoGTPase which act to degrade IκBα will not be able to activate NFκB in this scenario. In the control cells where wild-type IκBα is overexpressed, the NFκB is not markedly altered since wild-type IκBα can be degraded. This suggests that the Rho-NFκB mechanism for the inducible NFκB pathway is IκBα-mediated. In addition, this was not dependent on activation of Ras, a GTPase known to activate NFκB [41]. Nevertheless, some elements of the MAPK (mitogen-activated protein kinase) pathway, often downstream of Ras, may be involved. For example, the MAP3K1 {MEKK1 [MEK (MAPK/ERK (extracellular-signal-regulated kinase) kinase) kinase 1]} was found necessary for the NFκB activation induced by Rac1 and Cdc42 but not by RhoA [42].

On the other hand, one study on Swiss 3T3 cells found that TNFα-induced NFκB activation does not involve Rho. When TNFα was added to cells pretreated with the *Clostridium* Toxin B, a known Rho inhibitor, there was no difference in the TNFα-induced NFκB activation compared with cells without the Toxin B. The major limitation of this study, however, was that only immunofluorescence to detect NFκB component p65 was performed, without other assays such as EMSA (electrophoretic mobility-shift assay) or reporter assays. [43]

Rac1 [44–46] and Cdc42 [41] have been found respectively in different studies to be the main RhoGTPase that regulates NFκB. Activating mutants of Rac1-activated NFκB, which then increased cell proliferation via the expression of the cell-cycle regulator cyclin D1 [47]. Overexpression of non-phosphorylatable, and thus non-degradable, IκBα reduced the cyclin D1 promoter transcriptional activity. TNFα-stimulated Rac-activated NFκB is physiologically relevant, since it resulted in the expression of MMP9 [48] and cytokines [49].

In the case of TNFα-induced signalling, can the TNFα pathway stimulate the RhoGEFs? There are many ways this can occur. For example, it may occur through the protein kinase Cα, ERK activation, or through the activation of another receptor EGFR. [50,51]

### Adhesion related pathways

Integrins are important dimeric cell adhesion molecules, which can interact with extracellular matrix molecules and regulate cytoskeletal reorganization, motility, spreading and adhesion [52]. One important study [38] showed that β-integrin, a *bona fide* activator of Rho, could activate the Rho NFκB network. Activation of NFκB by integrins involves formation of focal adhesion complexes, the non-receptor syk kinase, and sometimes the ERK [53–55]. The extracellular molecules laminin-5 [56] or fibronectin [38] could engage α6/β4 integrins, resulting in Rac1 activation of NFκB. This mechanism is likely mediated by IκBα as shown by the experiments similar to those described in the previous section [57,58].

Yersinia invasin is a pseudotuberculous bacterial β1 integrin ligand [59]. It can cross-link mammalian integrins and regulate lymphocytic migration over collagen IV and fibronectin [60]. Beads coated with the integrin-binding domain of Yersinia invasin fused to maltose-binding protein offers a way to study the Rho–NFκB interaction. Phagocytosis, the ingestion of particles, is a integrin-mediated process [61]. During phagocytosis of the above beads by fibroblasts, β1, α4 and α5 integrins were the main membrane receptors for the beads [62]. Phagocytosis, but not the initial binding to beads, was RhoA dependent and resulted in NFκB activation. This pathway plays a role in regulation of IL1α and TNFα expression.

### Cytokine and stress-related pathways

RhoGTPases can be activated by stress, cytokines, vascular shear forces and UV radiation/DNA damage. The cytokines IL1α and IL1β bind to the same cellular receptor, IL-1RI, for IL1-mediated signalling. Upon receptor activation, IL-1R1 forms a heterodimer with IL-1RAcP (IL1 receptor accessory protein), which is essential for downstream signalling. Activation of NFκB can occur through different mechanisms implicating IL-1 receptor-associated kinases, IRAK-1 and IRAK-2. These kinases function as adapter proteins, recruiting TRAF6 to the receptor complex via an interaction with IL-1RAcP. Oligomerization of TRAF6 and subsequent formation of MAP3K7 (TAK1) and MEKK3 signalling complexes can activate via NIK, IKK-1/2, leading to NFκB activation [39,63]. There is currently no firm evidence that a MAPK such as JNK can regulate the Rho–NFκB activation. Sulciner et al. [64] showed that Rac1 regulated a cytokine-stimulated NFκB pathway. Overexpression of Rac1V12 has been shown to increase NFκB activity. This was not due to a autocrine mechanism, i.e., not due to a secreted factor downstream of Rac1V12 that stimulated NFκB, since the culture media supernatant from the transfected cells could not activate NFκB in cells not expressing Rac1V12. This effect of Rac1 was not shared by Cdc42, another RhoGTPase. This appears to contradict a study described above [41], but that study had used a different NFκB reporter (based on HIV) and was not performed using HeLa. Sulciner et al. showed that Rac1 operated downstream of IL-1β to activate NFκB [64], and that Rac1 operated downstream of Ras, consistent with other studies [65–67]. Similar to Rac1, Rac2 is a regulator of IL-1-induced NFκB activation, and this system controls IL-2 gene expression [63].

Cytokine effects can also manifest directly by changes in the GEFs. In breast carcinoma (MCF-7) cells stimulated with IL-1β, lipid rafts containing Vav1 and Rac1 could be detected. Cells overexpressing wild-type Vav1 showed the highest NFκB activity, followed by the GFP-transfected (control) cells, whereas cells with dominant negative Vav1 showed very low NFκB activity, similar to cells without IL-1β stimulation [40].

Under conditions of high shear stress in blood vessels, thrombin is a critical molecule for platelet aggregation. It has been shown to activate NFκB and hence regulate IL-8 expression. [68]. In pulmonary hypertension, Rac has been shown to increase thrombin-stimulated NFκB activity via the canonical IκBα pathway, resulting in the expression of a disease-causing tissue factor [69]. In the pulmonary vessel smooth muscles, the requirement for NFκB at the promoter site of HIF (hypoxia-inducible factor)1α is important for the Rac-regulated HIF1α expression [70].

Sensing of cellular stress requires NFκB activation [71]. UV can induce NFκB activation in many ways, including the generation of ROS as described previously. DNA damage, inducible by UV radiation or doxorubicin, also triggers NFκB activation [72]. Doxorubicin, an anticancer drug, is a anthracycline antibiotic which can exert its cytotoxic effect by intercalating between cellular DNA and effectively stopping DNA replication [73]. In HeLa cells, UV-activated NFκB, evidenced by down-regulation of IκBα, was suppressed by inhibition of RhoGTPase function [74]. These experiments were however based on using traditional Rho inhibitors, Cloistridium Cytotoxin B and lovastatin, and not by the specific interference with a RhoGTPase [74].

### Non-classical catenin pathway

Cell-to-cell adhesion complexes called adherens junctions contain proteins such as catenins, which are bound to membrane cadherins and intracellular actin. The p120 catenin is a recently discovered member of catenins bound to juxtamembrane cadherin and has regulatory roles on cell-to-cell adhesion [75]. It is a non-classical catenin in the sense that it does not bind directly to DNA. More recently, it was shown that p120 catenin can suppress inflammation in the skin. Compared with wild-type controls, p120 null mice, especially the older mice, had more inflamed skin. In the mutant mice, this process involved RhoGTPase-mediated activation of proinflammatory NFκB gene targets [76].

Normal p120 probably inhibits RhoA activation, since epidermis in p120 null mice showed a marked increase in active RhoA (total RhoA was unchanged), and active RhoA increased NFκB activation (Figure 1). IκB phosphorylation was enhanced in p120 null epidermis compared with wild-type, suggesting that the canonical NFκB activation pathway was activated.

The NFκB effect is physiologically important since targeting NFκB by dexamethasone also suppressed the epidermal hyperproliferation and inflammatory infiltrate in the skin grafts. The RhoA activity in skin grafts was not dependent on NFκB since dexamethasone did not reduce RhoA activity. It is not clear how RhoA in p120 wild-type mice was kept inactive and prevented from activating NFκB. The expression of a mutant p120 that could not interact with the cell adhesion molecule E-cadherin still down-regulated the p120 null-induced NFκB activation. Thus p120 does not need to bind to E-cadherin for the NFκB pathway to be quiescent. Overexpressing p120 that lacked its RhoGTPase-interacting domain could not suppress the p120 null activation of NFκB. This suggests that the interaction between p120 and RhoA is necessary to prevent RhoA from activating NFκB. Perhaps the binding of RhoA to p120 prevents the GEFs from switching RhoA to the GTP state. This is the first study showing the direct involvement of Rho–NFκB interaction in a disease phenotype.

### Immunological pathways

Given that NFκB is a master regulator in the immune system [23,71,77], RhoGTPases may, through NFκB, regulate many aspects of immunity, in particular innate immunity. TLRs (Toll-like receptors) are damage recognition receptors present on the cell surface that are responsible for detection of various insults and microbial agents, and the subsequent recruitment of other components of the immune system. Different TLRs have different affinities for ligands. For example, LPS (lipopolysarrcharide) produced by Gram-negative bacteria can stimulate the TLR4 receptors, whereas single-stranded unmethylated bacterial CpG DNA activate TLR9. Upon activation, TLR receptors activate intracellular signalling which involves several adaptor molecules such as the myeloid differentiation factor 88, resulting in the activation of the IKK complex and NFκB activation. This would induce the expression of IFN (interferon) and other immune related genes. Such signalling may involve NEMO and IκBα [78,79]. TLR2 was shown to trigger Rac1-, but not Cdc42-activated NFκB [80]. CpG DNA induced TLR9 mediated NFκB activation is inhibited by VavC, which is a mutant form of Vav that interferes with Vav1 function. Since Vav1 is a GEF for the Rac family of GTPases, it is plausible that Rac may be a mediator of CpG DNA-stimulated NFκB activation [81].

Upon stimulation by CD28, a TCR (T-cell receptor) costimulatory signal, Vav1, was shown to be an important GEF and activator of Rac1, which activated NFκB in an IKK-dependent fashion [82]. Vav-1 activated NFκB in diffuse large B-cell lymphoma [83], a very aggressive malignancy, can sometimes be treated with anti-CD40 therapy. Tumours resistant to such therapy are lacking in Vav-1 and cannot activate NFκB upon CD40 ligation. Therefore the presence of Vav1 can be potentially useful to identify responders in such therapies [83].

## INHIBITION OF IκBα DEGRADATION (NEGATIVE REGULATION OF NFκB)

In some instances, RhoGTPases have an inhibitory effect on NFκB. LPS and IFNγ were shown to activate NFκB in a RhoA-dependent fashion in C6 glioma cells [84]. In this case, RhoA had a negative regulatory effect on NFκB [84], in contrast to its positive regulatory role when UV was applied [74]. It is not clear if the discrepancy is due to a different cellular environment (central nervous system versus other types of cells) or due to different stimuli (LPS/IFNγ instead of other stimuli). The actual mechanistic difference is important in translational medicine since the LPS and IFNγ are triggers of nitric oxide and key to the regulation of inflammation in the central nervous system [84].

Other examples of inhibitory effects are provided by less studied members of Rho proteins. RhoH is a small (~21 kDa) G protein and a member of the Rac subfamily [85], which has no effect on actin or cytoskeletal organization [86]. Furthermore, RhoH is GTPase deficient [86] and remains in the GTP-bound activated state without cycling due to mutations in two highly conserved residues necessary for GTPase activity. RhoH was found to be a potent inhibitor of NFκB activation induced by other RhoGTPases [86]. TNFα-stimulated NFκB activity was almost completely suppressed by the expression of RhoH. Rac1 and RhoA-induced NFκB activity was also greatly suppressed by the expression of RhoH [86]. RhoH expression prolonged the detection of IκBα after the addition of TNFα, suggesting that it functions by reducing IκBα degradation. Although this may involve the competition with Rac1 and RhoA, the precise molecular mechanism is unknown.

Phorbolmyristate acetate, a potent activator of protein kinase C, was shown to down-regulate the RhoH mRNA levels in Jurkat cells [86], suggesting that this Rho protein can be transcriptionally regulated. This is intriguing given that the activities of more well-studied RhoGTPases (RhoA, Rac and Cdc42) are generally regulated by the action of GEFs and GAPs rather than by changing their own levels.

Overexpression of a RhoGTPase called RhoB [87] blocked NFκB activation. The inhibitory effect of Rac1 on NFkB activation has also been demonstrated in Nod2-dependent NFκB activation in the intestines [88]. The majority of the Rho proteins are modified by addition of a geranylgeranyl group, but RhoB is unique since it is present either as a geranylgeranylated (RhoB-GG) or a farnesylated (RhoB–F) form. These forms are functionally distinct. RhoB–F is a potent activator of NFκB, whereas much weaker activation is observed for other forms of RhoGTPases (i.e. RhoB-GG, RhoA and RhoC) [89]. When the CAAX box in RhoB was removed, or its palmitoylation sites mutated, the activation of the NFκB was greatly reduced. Overexpression of RhoB activated NFκB in HeLa, T47D and Cos-7 cells but not in NIH 3T3 cells. It is possible that the inhibitory effect of RhoB in NFκB [87] may be limited to murine fibroblasts. It has been suggested that RhoB, D, E and H are Rho proteins with inhibitory effects on gene transcription, whereas RhoA, G, Rac1,2 and Cdc42 may have activating effects [86] This model is overly simplistic since RhoA can also have inhibitory effects on NFκB activity [84].

## NUCLEAR TRANSLOCATION OF NFκB

In vascular endothelial cells [HUVEC (human umbilical-vein endothelial cells)], TNFα-stimulated NFκB was shown to regulate IL-8 expression. This activation of NFκB was found to be inhibited by *Clostridium difficile* toxin B. When RhoGTPase action was blocked, nuclear translocation was inhibited without suppression of IκB degradation. This implied that IκBa degradation does not necessarily serve as a signal for translocation of NFκB when the upstream signalling is regulated by RhoGTPase [90].

## PHOSPHORYLATION OF p65 TRANSACTIVATION DOMAIN OR p65 OVEREXPRESSION

Alteration of NFκB transcriptional activity has been attributed to the phosphorylation of the p65 subunit transactivation domain by a variety of kinases in response to different stimuli [91]. Apart from IκBα degradation, this is a major regulatory point of the NFκB pathway, and offers another route in which RhoGTPases can regulate this pathway. The two RhoGTPases that act on the phosphorylation of p65 are Rac1 and RhoB.

Staphylococcal peptidoglycan-stimulated NFκB activity is mediated by Rac1 in macrophages. This activation of NFκB is via IKK1/2, and phosphorylation of p65 at Ser536. This pathway is important for inflammation, mediating the release of COX2 (cyclo-oxygenase 2) and PGE_2_ (prostaglandin E_2_) [92]. Unfortunately, other studies of RhoGTPases on NFκB and COX2 expression did not examine p65 phosphorylation [93,94].

Bcr (B-cell receptor) signalling contributes to many important events in the immune system. Signalling mediated IKK activation requires upstream molecules such as the serine/threonine kinase PKC (protein kinase C)β, Tak1, some adapters and mucosal associated lymphoid 1, and these entities determine B-cell development [95]. However, this conventional model of Bcr does not incorporate the function of RhoGTPases. Suppression of RhoB activity by dominant-inhibitory mutants, or siRNA, was shown to block NFκB activation by Bcr and TSG (tumour-susceptibility gene)101. TSG101 is a molecule involved in endosomal sorting and trafficking. It was suggested that through an endosome pathway mediated by RhoB, Bcr and TSG101 can activate the NFκB [89]. The downstream effector of RhoB, ROCK1 (Rho-associated protein kinase 1), cooperates with RhoB to activate NFκB. Reduction of ROCK1 activity by genetic (dominant inhibitory ROCKI(KD) or pharmacological inhibitor Y-27632 blocks NFκB activation. NFκB activation by RhoB was not associated with increased nuclear translocation of p65, but it is suspected to be mediated via the phosphorylation of p65 transactivation domain [96].

## NUCLEAR LEVEL REGULATION OF NFκB

Nuclear Vav1 is a component of active transcriptional complexes that contain NFκB [97]. The nuclear localization of Vav1 occurs in a stimulation-dependent manner and requires two structural components: a COOH-terminal SH3 (Src homology 3) domain and a nuclear localization sequence within the pleckstrin homology domain of Vav1. However, it is not clear whether this function of Vav1 is at all related to its RhoGEF function.

Rac3 can also activate NFκB through a nuclear mechanism, serving as a nuclear coactivator for NFκB-dependent transcription. There is competition between transcription factors in different pathways for the same coregulators. For example, the transcriptional effects of glucocorticoid receptors and NFκB depend critically on the abundance of shared nuclear coregulators such as Rac3 [98].

## RHOGTPASE NON-CANONICAL NFκB INTERACTIONS

Other than the canonical pathway, RhoGTPases can affect NFκB in processes that have been much less investigated. For example, in colorectal DLD-1 cells, NFκB activation is not dependent on IκBα degradation or NFκB nuclear translocation. Instead, Rac1 was involved in the expression of p50/p105, part of the non-canonical NFκB [99]. Angiotensin II stimulates the phosphorylation of p65 at Ser^536^ through the mediation of Rac and RhoA, which requires the NIK/MEKK14 complex and the association of p65 with NIK. These events triggered NFkB2 p100 processing and p52 nuclear accumulation and the expression of IL-6 [100].

Another example of the non-canonical pathway comes from tumour biology. The GEF Dbl has been shown to regulate RhoA, Rac, Cdc42 to activate NFκB, resulting in cyclin D1 expression and malignant transformation [101]. Rac-mediated activation of NFκB, but not the RhoA/Cdc42 activation of NFκB, is mediated by IKK2 [101]. NFκB is linked to EMT (epithelial–mesenchymal transition), and Rho and Rac have been implicated in EMT in tumours [102]. RhoGTPases may therefore be involved in activation of the non-canonical NFκB that regulates EMT.

## OTHER WAYS RHO-GTPASES INTERACT WITH NFκB

Apart from increasing RhoGTPase activity, NFκB can be regulated by subcellular localization or targeting of RhoGTPase and IκBα, or by alternative splicing of RhoGTPase. An unusual way for Rho to activate NFκB is exemplified by the role of Rac as a docking molecule. Rac is important for localizing the E3-ligase Cullin and IκBα to membrane ruffles. In ruffles, IκBα can be degraded with consequential activation of NFκB [103].

In another scenario, the linking or scaffolding function may not be performed by the RhoGTPase but by a GEF. Vav proteins have several protein-binding domains that link cell-surface receptors to downstream signalling proteins. Vav1 is expressed exclusively in haematopoietic cells but Vav2 and Vav3 are more broadly expressed [104]. Vav1 and Vav3, but not Vav2, can facilitate NFκB-dependent transcription. A Vav-1-deficient Jurkat T-cell line not only has reduced NFκB activity, but also has defects in the T-cell antigen receptor signalling [105].

Matos et al. [106,107] investigated an alternatively-spliced variant of Rac1 called Rac1b, often found in colon or breast cancers. Mouse 3T3 cells express Rac1 constitutively but not Rac1b [106], and in overexpression studies, phosphorylation of IκBα was found to be higher in the active forms of Rac1 and Rac1b compared with the dominant negative forms. Expression of Rac1 and Rac1b induced nuclear localization of p65. Unlike with oncogenic Ras, activating mutations of Rac have not been reported in tumours. These findings [106] suggest that alternative splicing of Rho may be more important in oncogenesis than point mutations. Can RhoGTPases be regulated by covalent modifications [108] in physiology and signal to NFκB? Apart from farnesylation, the effect of other covalent modifications, such as ubiquitylation and degradation of RhoGTPases, on NFκB activation remains unclear [109]. The reason for one specific RhoGTPase instead of another RhoGTPase activating the NFkB is often unknown. This should be the focus of more research in the future.

## CONCLUDING REMARKS

**Figure 2:**
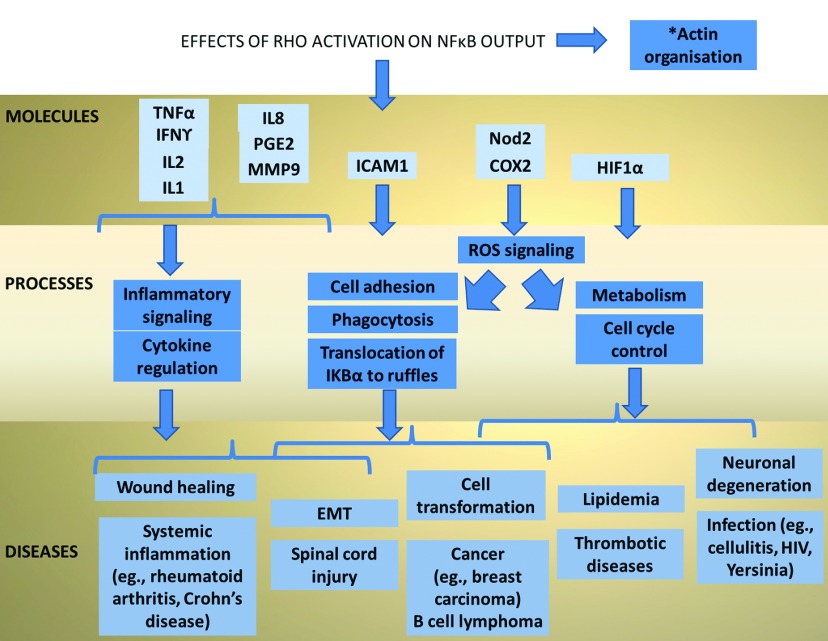
Biological process and diseases affected by Rho NF*k*B signalling This figure shows the medical significance of the Rho NFκB signalling, the therapeutic targets shown in the top row could be useful in a variety of human diseases shown on the bottom row. IL1 [76], interleukin 1; IL8 [90], interleukin 8; ICAM1 [46], intercellular cell adhesion protein 1; COX2 [94], cyclo-oxygenase; MMP [48)], matrix metalloproteinase; TNFα [49], tumour necrosis factor α; IFNγ [48], interferon gamma; Nod2 [88], nucleotide-binding oligomerization domain-containing protein 2; HIF1α [70], hypoxia-induced factor; EMT [102], epithelial–mesenchymal transition; ROS, reactive oxygen species. Relevant diseases include skin inflammation or infection (Cellulitis) [76] Viral infections, e.g., HIV [113], Yersinia [59]; Systemic inflammation, e.g. Rheumatoid arthritis, Crohn's disease [114]; Thrombotic diseases [68]; Breast carcinoma [107]; Haematological malignancies, e.g., B-cell lymphomas [83]; Metabolic diseases, e.g., hyperlipidemia [94]; and Spinal cord injury [115].

Multiple different aspects of cellular function are regulated by the interaction of RhoGTPases and NFκB signalling. Rac1 and cdc42 have a facilitatory role in NFκB, RhoH is associated with a inhibitory role in NFκB activation, whereas RhoA and RhoB can have either a positive or negative regulatory role depending on the context. This network is governed by the intracellular processes as well as those involving interactions with extracellular matrix. Since the upstream triggers include cytokines, catenin, integrins and UV, the outcome of the RhoGTPase–NFκB interaction can determine the important processes that govern cell fate. More importantly, there are therapeutic implications in human diseases that may be exploited by targeting this pathway (Figure 2). Pedersen et al. have introduced a new tool in RhoGTPase research: a model of primary keratinocytes (skin epithelial cells) from adult mice, which is an inducible RhoA knock out system [110]. Ultimately, *in vivo* animal models lacking Rho [111] are important for studying the native Rho functions and the associated NFκB activity under basal and stimulated conditions. The complexity of the Rho-dependent NFκB activation is immense, since different Rho proteins can crosstalk [112]. The actual mechanisms of NFκB signalling are very cell-specific, and it is uncertain how much one can extrapolate from the findings of individual studies. Table 2 shows the cell types in which the RhoGTPase NFκB relationship has been evaluated. Nevertheless, our understanding of the less-studied members of the Rho family seems rapidly increasing. With advances in cellular assays, bioimaging, computational tools and sophisticated animal models, research will yield details of the Rho-NFκB networks. It is somewhat disappointing that successful experimental inhibition of NFκB in animal models has not yet been replicated in human diseases. Combined targeting of RhoGTPase and NFκB could perhaps achieve improved therapeutic effects in a number of human ailments.
